# Mayaro virus detection in the western region of Pará state, Brazil

**DOI:** 10.1590/0037-8682-0055-2020

**Published:** 2021-03-22

**Authors:** Cassiano Junior Saatkamp, Luís Reginaldo Ribeiro Rodrigues, Andrew Mairom Nogueira Pereira, João Alberto Coelho, Rose Grace Brito Marques, Victor Costa de Souza, Valdinete Alves do Nascimento, Jamille Gomes dos Santos Saatkamp, Felipe Gomes Naveca, Regina Maria Pinto de Figueiredo

**Affiliations:** 1 Rede BIONORTE, Programa de Biodiversidade e Biotecnologia, Santarém, PA, Brasil.; 2 Universidade Federal do Oeste do Pará, Campus Tapajós, Laboratório de Genética e Biodiversidade, Santarém, PA, Brasil.; 3 Laboratório Santos, Santarém, PA, Brasil.; 4 Universidade do Estado do Pará, Santarém, PA, Brasil.; 5 Instituto Esperança de Ensino Superior, Santarém, PA, Brasil.; 6 Secretaria Municipal de Saúde, Divisão de Vigilância em Saúde, Santarém, PA, Brasil.; 7 Fundação Oswaldo Cruz, Instituto Leônidas e Maria Deane, Manaus, AM, Brasil.; 8 Fundação de Medicina Tropical Doutor Heitor Vieira Dourado, Manaus, AM, Brasil.

**Keywords:** Arbovirus, Mayaro virus, Molecular diagnosis, Real-Time Polymerase Chain Reaction

## Abstract

**INTRODUCTION:**

Mayaro virus (MAYV) was found in Pará state, Brazil, in 1955. Since then, sporadic outbreaks have occurred in different regions of the country.

**METHODS:**

Serum sample were collected from 49 individuals in 2016 and were initially tested for dengue virus (DENV) by real-time (RT) polymerase chain reaction (PCR). DENV-negative samples were tested for MAYV and Oropouche virus (OROV) by multiplexed RT quantitative PCR.

**RESULTS:**

All samples were negative for DENV and OROV, but MAYV was detected in four samples.

**CONCLUSIONS:**

Differential diagnoses of acute febrile syndrome are required, especially in regions where several arboviruses with similar clinical manifestations are endemic.

Arboviruses represent a threat to public health in several countries. In Brazil, the most important arboviruses with the potential for dissemination are dengue, chikungunya, and Zika, in addition to the yellow fever virus^1^. However, other neglected, emerging, or re-emerging arboviruses, such as those belonging to the *Togaviridae* and *Peribunyaviridae* families, are also important. The Mayaro virus (MAYV), part of the *Togaviridae* family, belongs to the group of alphaviruses called the Semliki Forest Complex, which includes the Una, Bebaru, chikungunya, Getah, Ross River, Igbo-Ora, O'nyong-Nyong, Sagyama, and Semliki Forest viruses^2^. MAYV infection causes a chikungunya-like febrile syndrome with arthralgia/arthritis lasting for 2 weeks, which may likely be misdiagnosed as dengue and chikungunya fever due to their similarities^3^. The symptoms range from mild to severe and may present with headache, rash, myalgia, arthralgia in the large joints, and sometimes arthritis. MAYV can produce severe complications, such as intermittent fever, neurological complications, myocarditis^4^.

MAYV transmission occurs in the enzootic cycle, mainly involving wild *Haemagogus janthinomys* mosquitoes and mammalian vertebrate hosts. However*,* the virus also uses vectors of the genus *Aedes*, as observed for yellow fever, through genetic mutations of the virus. Moreover, it can spread in urban regions through contaminated birds and humans^5^.

Due to the similarity of the clinical aspects of MAYV infection with other arbovirus infections, the correct diagnosis of this disease may be difficult, hindering the magnitude and frequency of outbreaks until they are considered sporadic since the MAYV true infected patient may be misdiagnosed as a dengue or chikungunya one. The method most commonly used for MAYV detection is antibody serology. However, this method may be affected by the occurrence of cross-reactivity with other members of the *Alphavirus* genus, such as the Semliki Forest, Getah, Una, and chikungunya viruses^6^.

Most of the cities in the northern region of Brazil are close to forested areas. Even populations in the largest urban areas have experienced Mayaro fever outbreaks over the last decade, such as Manaus (Amazonas) in 2007^7^ and Ananindeua, Vigia, and Acará (Pará, PA), which reported laboratory-confirmed MAYV cases in 2019^8^. Permanent epidemiological and entomological studies should be performed to determine MAYV endemic areas and the risk of transmission to human hosts, especially in areas where the disease has already been confirmed^5^. In this study, we detected MAYV in patients with acute febrile syndrome in western Pará state using molecular methods.

The municipality of Itaituba **(**4° 16' 9'' S; 55° 59' 23'' W) is situated in the southwestern region of Pará state. Itaituba has approximately 97,343 inhabitants and is an important economic hub for gold mining, agribusiness, and forestry products. The municipality of Alenquer (01° 56' 30″ S; 54º ° 44' 18″ W) is situated in the Lower Amazon region, with a population of nearly 56,480.

The patients enrolled in this study (n = 49) visited the public health units of the municipalities of Itaituba and Alenquer in 2016 with an acute febrile clinical condition, and blood samples were collected during the acute phase of infection. Subsequently, sera samples were sent to the reference hospital for infectious diseases-Tropical Medicine Foundation Doctor Heitor Vieira Dourado (FMT-HVD), Manaus, Amazonas state. Ribonucleic acid (RNA) was extracted using the QIAamp Viral RNA Mini Kit (QIAGEN Biotechnology, SP Brazil), following the manufacturer’s instructions, and used for a semi-nested multiplex polymerase chain reaction (PCR) protocol searching for dengue virus (DENV) RNA^9^. Samples negative for DENV were subjected to multiplex real-time quantitative PCR (RT-qPCR) for MAYV and Oropouche virus (OROV)^10^. This study was approved by the Human Research Ethics Committee of Hope Institute for Higher Learning - IESPES (Protocol No. 3.149.097).

All samples were negative for DENV (n = 49). Further, all samples were negative for OROV, but four (8.2%) samples were positive for MAYV, with a cycle threshold Ct (cycle threshold) between 34.0 and 36.9 (two males and two females aged between 12 and 45 years). The patients reported fever, headache, myalgia, nausea, and rash, with only one patients presenting with severe arthralgia. Two cases had acute febrile syndrome for 4 days, while the other two cases had acute febrile syndrome for more than 5 days after the initial onset of symptoms (Table 1). Fischer et al. (2020)^11^ showed that in previous studies (both for surveillance of malaria cases), MAYV infection was detected in approximately 10.8% of the samples tested, and the distribution of cases was similar for both females and males. Thus, these data are similar to the observations of the present study.

**TABLE 1:** Epidemiological and clinical data from MAYV infected patients in the Western Pará region.

			Clinical Features						
Sample Code	Sex/Age (years)	*d*	Fever	Headache	Myalgia	Rash	Nausea	Arthralgia	Retroorbital pain
ALT/11	F/18	+ 5	NR	NR	NR	NR	NR	NR	NR
ITB/10	M/12	4	Yes	Yes	Yes	Yes	Yes	No	No
ITB /15	F/45	+ 5	Yes	Yes	Yes	Yes	Yes	Yes	Yes
ITB /17	M/15	4	Yes	Yes	Yes	Yes	No	No	No

**M:** male; **F:** female; ***d:*** days of symptom onset; **NR:** not reported.

Our results are in accordance with the data found in the literature. Lorenz et al. (2017)^12^ characterized MAYV infection by nonspecific symptoms that persist for 3 to 5 days, which can be confused with DENV, OROV, chikungunya, and other arbovirus infections and can affect individuals of all ages.

MAYV was first isolated in 1954 from forest workers in Trinidad. In Brazil, the first outbreak of Mayaro fever was reported in 1955 in the Guamá River region, Pará state. A huge outbreak occurred in the municipality of Belterra, western Pará state, in 1978. Since then, sporadic outbreaks have been reported, mainly in the Brazilian Northern and Midwestern regions^13^.

It is important to mention that causative agents of severe acute fever cases remain undiagnosed in most parts of the Amazon region due to the inherent limitations such as the large territorial area, low population density, and the logistical and infrastructure deficiencies of public health services in the region^14^. Moreover, the misdiagnosis of such disease agents can be strengthened, because several arboviruses endemic to that region (e.g. Dengue, Zika, Oropouche, and Chikungunya) leads to clinical manifestation of similar symptoms.

In this study, we observed cases that had symptoms for 4 and more than 5 days. It is important to highlight the difficulty of obtaining clinical and epidemiological data. Most epidemiological questionnaires are not fully completed. However, there are a limited number of clinicians, particularly in the most distant municipalities.

Through RT-qPCR, we found new MAYV cases that were undetected in routine diagnostics by public health services throughout the Amazon region. Since the clinical features may result in an inconclusive diagnosis, we reiterate the need for differential diagnoses, especially in regions where several arboviruses with similar clinical manifestations are endemic. It will then become possible to define the real importance of MAYV and other arboviruses to Brazilian public health.
